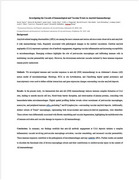# Investigating the Cascade of Immunological and Vascular Events in Amyloid Immunotherapy

**DOI:** 10.1002/alz70861_108872

**Published:** 2025-12-23

**Authors:** Xavier Taylor

**Affiliations:** ^1^ Eli Lilly and Company, Indianapolis, IN USA

## Abstract

**Background:**

Amyloid‐related imaging abnormalities (ARIA) are among the most common and serious adverse events observed in anti‐amyloid‐β (Aβ) immunotherapy trials, frequently associated with pathological changes in the cerebral vasculature. Cerebral amyloid angiopathy (CAA) represents a primary site of antibody engagement, triggering vascular inflammation and increasing susceptibility to microhemorrhages. Emerging evidence highlights the role of perivascular macrophages and infiltrating immune cells in modulating vascular permeability and injury. However, the downstream molecular cascades initiated by these immune responses remain poorly understood.

**Methods:**

We investigated immune and vascular responses to anti‐Aβ (3D6) immunotherapy in an Alzheimer’s disease (AD) mouse model of microhemorrhage. Histology, RNA in situ hybridization, and NanoString digital spatial proteomics and transcriptomics were used to define cellular interactions and gene expression changes surrounding vascular amyloid deposits.

**Results:**

In the present study, we demonstrate that anti‐Aβ (3D6) immunotherapy induces immune complex formation at CAA sites, leading to smooth muscle cell loss, blood–brain barrier disruption, and extravasation of plasma proteins, coinciding with hemosiderin‐laden microhemorrhages. Digital spatial profiling further reveals robust recruitment of perivascular macrophages, monocytes, and peripheral immune cells—including T and B lymphocytes—surrounding vascular amyloid deposits. Additionally, distinct subsets of Trem2⁺ macrophages, representing both tissue‐resident and monocyte‐derived populations, were identified. These subsets were differentially associated with fibrotic remodeling and vascular degeneration, highlighting the multifaceted roles of immune activation and vascular damage in response to Aβ immunotherapy.

**Conclusions:**

In summary, our findings establish that anti‐Aβ antibody engagement at CAA deposits initiates a complex inflammatory cascade involving perivascular macrophage activation, vascular remodeling, and increased vascular permeability. These immune responses contribute to the pathogenesis of microhemorrhages and may underlie ARIA. Further studies are needed to elucidate the functional roles of diverse macrophage subsets and their contributions to cerebrovascular injury in the context of Aβ immunotherapy.